# *Legionella* spp. isolation and quantification from greywater

**DOI:** 10.1016/j.mex.2015.11.004

**Published:** 2015-11-10

**Authors:** Sara Rodríguez-Martínez, Marina Blanky, Eran Friedler, Malka Halpern

**Affiliations:** a Department of Evolutionary and Environmental Biology, Faculty of Natural Sciences, University of Haifa, Haifa, Israel; b Faculty of Civil and Environmental Engineering, Technion, Haifa, Israel; c Department of Biology and Environment, Faculty of Natural Sciences, University of Haifa, Oranim, Tivon, Israel

**Keywords:** Greywater, *Legionella* isolation, Pre-filtration, Acid-thermic treatment, Culture, ISO 11731:1998

## Abstract

*Legionella*, an opportunistic human pathogen whose natural environment is water, is transmitted to humans through inhalation of contaminated aerosols. *Legionella* has been isolated from a high diversity of water types. Due its importance as a pathogen, two ISO protocols have been developed for its monitoring. However, these two protocols are not suitable for analyzing *Legionella* in greywater (GW). GW is domestic wastewater excluding the inputs from toilets and kitchen. It can serve as an alternative water source, mainly for toilet flushing and garden irrigation; both producing aerosols that can cause a risk for *Legionella* infection. Hence, before reuse, GW has to be treated and its quality needs to be monitored. The difficulty of *Legionella* isolation from GW strives in the very high load of contaminant bacteria. Here we describe a modification of the ISO protocol 11731:1998 that enables the isolation and quantification of *Legionella* from GW samples. The following modifications were made:

• To enable isolation of *Legionella* from greywater, a pre-filtration step that removes coarse matter is recommended.
• *Legionella* can be isolated after a combined acid-thermic treatment that eliminates the high load of contaminant bacteria in the sample.

## Graphical abstract

**Figure:**
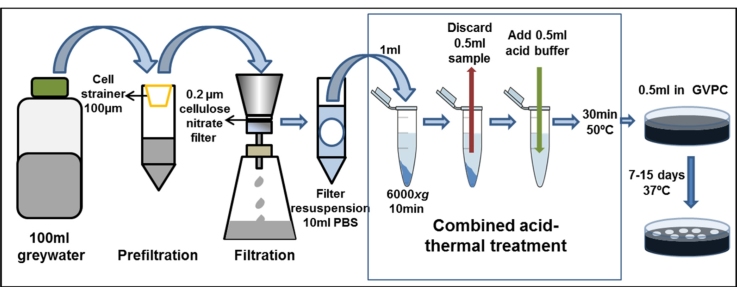

## Method details

There are several ISO methods for *Legionella* isolation from water [1], [2]. However, none of them is suitable for *Legionella* isolation from GW samples due to the fact that GW is contaminated with a very high bacterial load. Thus, almost no data can be found regarding *Legionella* presence in GW.

Here we describe a modification of the ISO 11731:1998 protocol for *Legionella* isolation from GW. Our modified protocol allows the isolation of *Legionella* on GVPC selective *Legionella* medium (glycine–vancomycin–polymyxin–cycloheximide medium, Beckton Dickinson GmbH, Heidelberg, Germany) without the massive bacterial contamination that develops on the media when ISO 11731:1998 is applied.

### Pre-filtration of the greywater sample

**Fig. 1:**
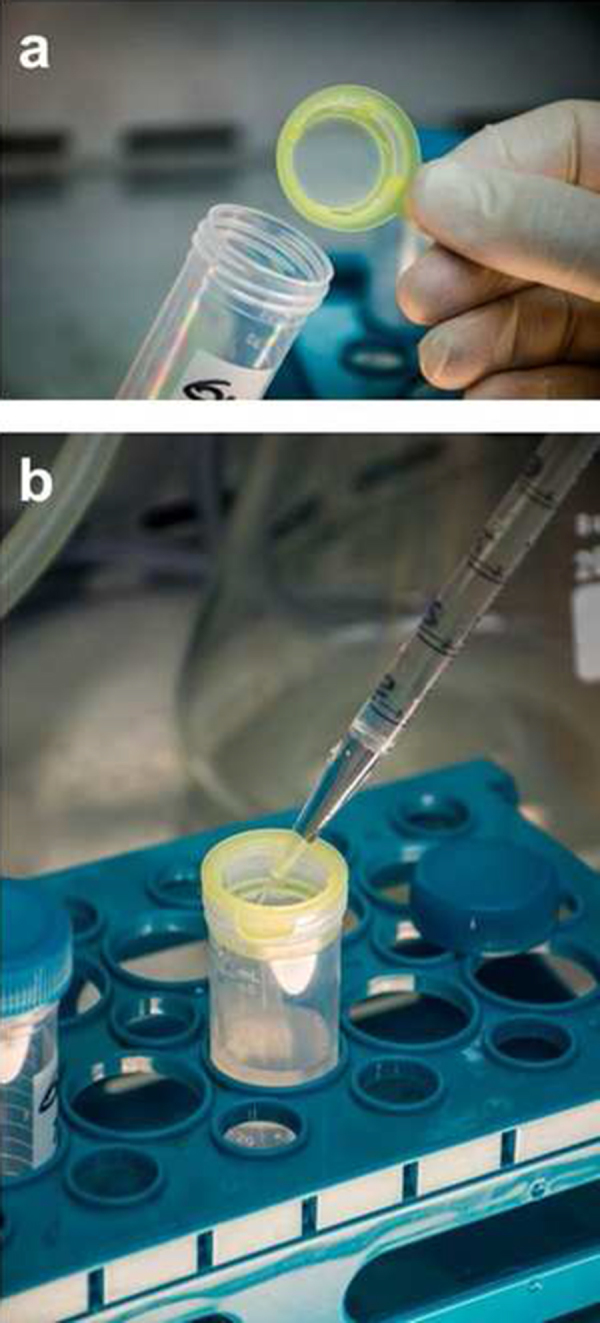
(a) A 100 μm pore size cell strainer. (b) Pre-filtration of the greywater samples trough the 100 μm pore size cell strainer.

Filter a 100 ml GW sample (pre-filtration) to remove coarse matter, using a 100 μm pore size cell strainer (Becton Dickinson, USA) placed in one 50 ml tube (two 50 ml tubes are needed) (Fig. 1).

### Filtration of the GW sample

**Fig. 2:**
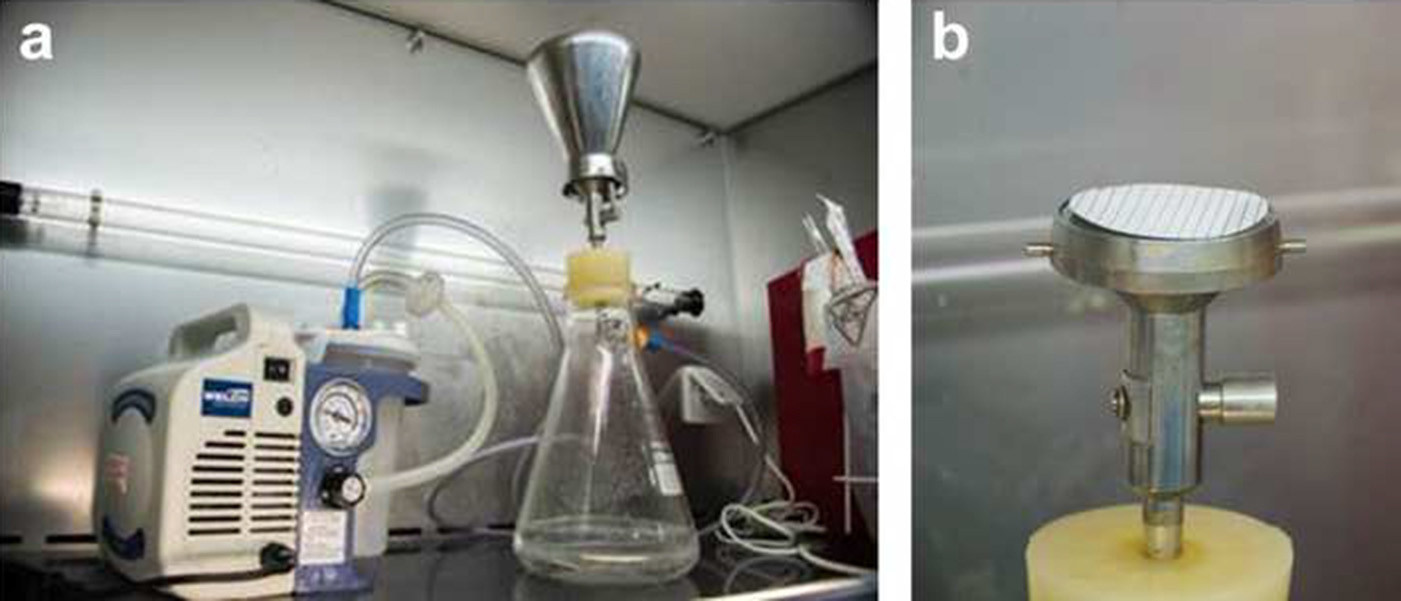
(a) A vacuum filtration system set-up: the filtration system comprised a stainless steel filter holder and its funnel attached by a rubber bung to a Buchner flask. The flask is connected by a rubber tube to a 2511 Dry Vacuum Pump (WELCH, Germany). (b) Zoom in on the 0.2 μm cellulose nitrate filter which is placed on the filter holder of the filtration system.

The 100 ml pre-filtered GW sample is filtered again through a 0.2 μm cellulose nitrate filter using a vacuum filtration system attached to a 2511 Dry Vacuum Pump (WELCH, Germany) (Fig. 2).

### Bacteria resuspension in PBS

**Fig. 3:**
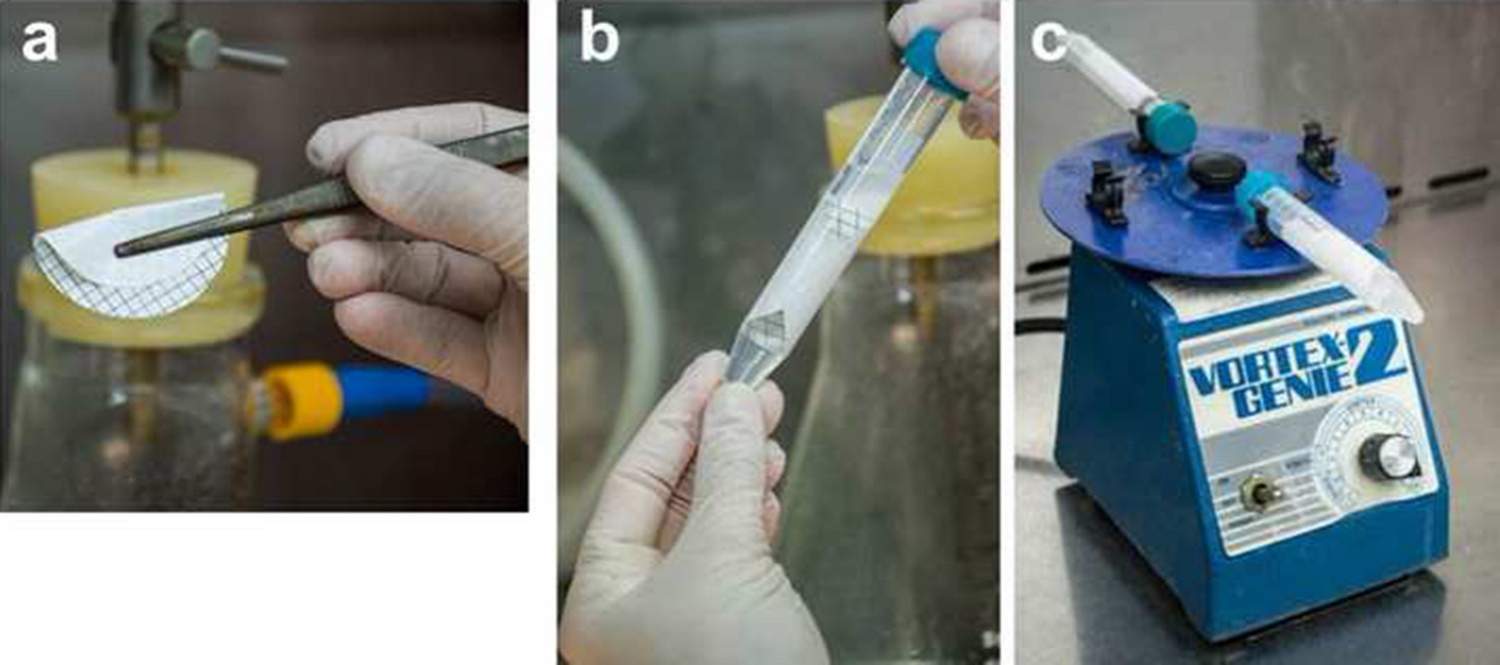
(a) The filter is folded before inserting it into the 10 ml PBS solution in a tube. (b) The filter inside the tube. The filter upper part must face the inside of the tube and it should overlap as less as possible. (c) Tubes are vortexed for 10 min using a 10 ml tube adaptor.

After filtration, the filter is placed into 10 ml phosphate buffered saline (PBS, pH 7.4 ± 0.2; NaCl 0.14 M; KCl 2.7 mM; Na_2_HPO_4_ 10 mM, KH_2_PO_4_ 1.8 mM) and vortexed for 10 min (Fig. 3).

### Combined acid–thermal treatment

Each sample is then subjected to a combined acid–thermal treatment as follows: 1 ml of the sample is centrifuged at 6000 × *g* for 10 min. For the acid treatment, 0.5 ml of the supernatant is replaced with 0.5 ml of acid buffer (HCl 27 mM; KCl 173 mM, pH 2.2). The sample is then vortexed and immediately subjected to thermal treatment for 30 min at 50 °C.

### GVPC *Legionella* media inoculation

Following the ISO 11731:1998 recommendations, two 0.5 ml sub-samples are plated on a GVPC *Legionella* selective media immediately after the thermal treatment. The plates are incubated at 37 °C. Presumptive *Legionella* colonies are counted after 7 and 15 days of incubation.

### *Legionella* identification

The presumptive *Legionella* colonies are then identified using a *Legionella* latex test (Oxoid, Basingstoke, UK). This test allows separate identification of *Legionella pneumophila* serogroup 1 and serogroups (2–14) and the detection of seven other *Legionella* species (*L. longbeachae* serogroups 1 and 2, *L. bozemanii* serogroup 1, *L. dumoffii*, *L. gormanii*, *L. jordanis*, *L. micdadei* and *L. anisa*).

### Method validation: efficiency of the *Legionella* isolation protocol and limit of detection (LOD)

We used the method described above to successfully isolate and quantify *Legionella* along a one year GW monitoring campaign. The results of this study have been already published [3]. Briefly, a total of 16 greywater samples were analyzed. *Legionella* was isolated from 81% of the samples, with a mean of 1.2 × 10^5^ cfu/l. Details about the efficiency and the limit of detection of this method can also be found in the mentioned publication. This method is highly aggressive, so the recovery rates of *Legionella* were very low (2.5%, SD = 1.5%) and the LOD established from this average recovery rate was 4.0 × 10^3^ cfu/l. Nevertheless, the results were consistent. It should be noted that this modified methods is the only way to isolate *Legionella* from GW, as using the current ISO protocols does not allow the isolation of this bacteria.

### Recommendations

This method is highly aggressive for the sampled bacteria, including *Legionella*. For that reason, the LOD of the method is high and the efficiency of *Legionella* isolation is low. We recommend using this method only with problematic samples in which *Legionella* can’t be isolated using the methods described in the ISO protocols 11731:1998 and 11731-2:2004 [1], [2] due to massive contamination with other bacterial species.
